# Heat-Killed *Bifidobacterium bifidum* B1628 May Alleviate Dextran Sulfate Sodium-Induced Colitis in Mice, and the Anti-Inflammatory Effect Is Associated with Gut Microbiota Modulation

**DOI:** 10.3390/nu14245233

**Published:** 2022-12-08

**Authors:** Cuijiao Feng, Weiqin Zhang, Tao Zhang, Qiuwen He, Lai-Yu Kwok, Yan Tan, Heping Zhang

**Affiliations:** 1 Inner Mongolia Key Laboratory of Dairy Biotechnology and Engineering, Inner Mongolia Agricultural University, Hohhot 010018, China; 2 Key Laboratory of Dairy Products Processing, Ministry of Agriculture and Rural Affairs, Inner Mongolia Agricultural University, Hohhot 010018, China; 3 Key Laboratory of Dairy Biotechnology and Engineering, Ministry of Education, Inner Mongolia Agricultural University, Hohhot 010018, China; 4 Shenzhen Xbiome Biotech Co., Ltd., Shenzhen 518000, China

**Keywords:** inflammatory bowel disease, gut microbiota, postbiotics, *Bifidobacterium bifidum*, heat-killed, anti-inflammation

## Abstract

Inflammatory bowel disease (IBD) is a chronic inflammatory disease associated with gut dysbiosis. This study aimed to investigate the effects of heat-killed *Bifidobacterium bifidum* B1628 (HB1628) in dextran sulfate sodium (DSS)-induced colitis in mice. The following three mouse groups were included (n = eight per group): NC (normal control), DSS (colitis), and HB1628 (colitis and postbiotic). The mice in the DSS group showed significant weight loss and histological damage, developed bloody diarrhea, scored high in the disease activity index (DAI), and exhibited increases in pro-inflammatory cytokines (interleukin [IL]-1β, IL-6, and tumor necrosis factor [TNF]-α) and decreases in an anti-inflammatory cytokine (IL-13) in the serum. These changes were accompanied by gut microbiota modulation in colitis mice (decreases in *Rikenellaceae* and *Eubacterium*; increases in *Peptostreptococcaceae*, *Bacteroides vulgatus*, and *Parasutterella excrementihominis*). The HB1628 group had lower DAIs, histology scores, and serum levels of pro-inflammatory cytokines (IL-1β and TNF-α), but higher levels of an anti-inflammatory cytokine (IL-13), compared with the DSS group, suggesting a less severe inflammatory state after the HB1628 intervention. Additionally, HB1628 improved DSS-induced gut dysbiosis, which is evidenced by increases in intestinal beneficial bacteria, such as *Lactobacillus*, and decreases in known unfavorable taxa in IBD, e.g., *Porphyromonadaceae*, *Subdoligranulum*, *Lachnospiraceae bacterium* 3_1_46FAA, and *Alistipes indistinctus*. Functional metagenomics revealed three significantly enriched metabolic pathways in the HB1628 group (namely, the aerobic respiration I [cytochrome c] pathway and the superpathways of L-phenylalanine biosynthesis and L-tryptophan biosynthesis, respectively). In conclusion, our results showed that HB1628 effectively improved the inflammation state and tissue damage in DSS-induced colitis mice, and the symptom relief effect was accompanied by obvious gut microbiota remodulation.

## 1. Introduction

Inflammatory bowel disease (IBD) is an immune-mediated intestinal disease, and its etiology involves a number of factors, including environmental triggers, genetics, microbial gut dysbiosis, and host immunity [1]. Currently, the clinical management of IBD mainly includes drug treatments and/or surgery, and the long-term treatment target is the absence of disability and normalized health-related quality of life [2]. However, the available treatments have some drawbacks, particularly the side effects of drugs and post-surgery complications. In-depth studies of the human gut microbiota using different sequencing technologies have gradually revealed its role in various acute and chronic diseases. A large body of evidence indicates that IBD is related to gut dysbiosis, which is characterized by a reduction in beneficial microorganisms and an overgrowth of pathogenic bacteria in the intestinal microenvironment [3]. Therefore, microbial interventions targeting the gut microbiota have gradually become a hot area of research for their therapeutic potential in alleviating IBD [4].

Fecal microbiota transplantation (FMT) is the transplantation of fecal microbiota from pathogen-free healthy donors to patients’ gastrointestinal tracts, aiming to reestablish a healthy gut microbiota and restore its beneficial functions [5]. This method has demonstrated clinical efficacy in treating IBD [6]. Tan et al. reviewed papers on the clinical application of FMT up to 8th November 2021, and studies have found that fresh FMT could improve clinical remission rates [7]. However, the administration method, the donor’s microbiota composition, and the choice of strains are all factors that influence the clinical responses of FMT in subjects with IBD [8]. Probiotics could lower inflammation, improve barrier functions, and restore a relatively healthier gut microbiota from its original dysbiotic state. Therefore, the American Gastroenterological Association (AGA) Institute recommends the use of probiotics in the context of clinical trials for adults and children with Crohn’s disease and ulcerative colitis (UC) [9]. A systematic review of 22 studies published from 1997 to 2019 pointed out that combining standard treatments with probiotics might be an option to achieve remission in patients with active UC [10]. However, some studies did not observe significant clinical effects of probiotics on improving IBD. A study found that the probiotic formulation of *Lactobacillus acidophilus* La-5 and *Bifidobacterium animalis* subsp. *lactis* BB-12 exerted no significant clinical benefits in alleviating the progression of colitis in patients with IBD [11], and, indeed, adverse events, such as diarrhea and nausea, were observed when *Escherichia coli* Nissle 1917 was applied [12]. It is possible that the non-responsiveness and even adverse effects could be related to the probiotic strain specificity and/or the actual activity/interaction of live bacteria with the host. Both FMT and probiotic intervention rely on introducing live bacteria into patients’ intestines, so the biosafety risk should not be ignored.

A postbiotic is defined as the “preparation of inanimate microorganisms and/or their components that confers a health benefit on the host” [13]. An increasing number of studies have demonstrated the promising clinical effects of postbiotic administration in managing various diseases. For example, a clinical trial has confirmed that heat-killed *Bifidobacterium bifidum* MIMBb75 could relieve irritable bowel syndrome [14]. Non-viable *Lactobacillus reuteri* DSMZ 17648 actively controlled *Helicobacter pylori* infections [15]. A few other pre-clinical and clinical studies have demonstrated the safe and remarkable effects of administering heat-killed *Akkermansia muciniphila* for improving multiple metabolic indices in overweight/obese insulin-resistant patients, which were comparable with those of supplementing live *Akkermansia muciniphila* [16,17]. Postbiotics offer some advantages over probiotics, particularly their storage stability and biosafety due to their non-viability. When it comes to inflammatory gastrointestinal diseases, such as IBD, viable bacteria may aggravate inflammation by activating innate immunity through microbe-associated molecular patterns [18] and, consequently, worsen the symptoms. Postbiotics have shown a variety of biological activities, including effects on anti-inflammation, anti-hypertension, anti-oxidation, immune regulation, serum cholesterol reduction, and inhibition of abnormal cell proliferation [19]. At present, research on postbiotic function is still in its infancy, and further studies would be needed to unveil other beneficial effects and clarify the probiotic mechanisms of postbiotics.

*Bifidobacterium bifidum* B1628 was isolated from the fecal sample of a healthy infant in the pastoral area of Haibei Prefecture, Qinghai Province. The strain showed good acid and bile salt tolerance. Additionally, the bacterial metabolites were rich in ascorbic acid, caffeic acid, melatonin, and isobutyl ketone; thus, they exerted anti-oxidation, anti-inflammatory, and gastrointestinal conditioning effects through untargeted metabonomic analysis (data are unpublished). This study aimed to investigate the effects of administering heat-killed *Bifidobacterium bifidum* B1628 (HB1628) in a mouse dextran sulfate sodium (DSS)-induced colitis model, particularly from the perspectives of immune regulation and gut microbiota modulation.

## 2. Materials and Methods

### 2.1. Preparation and Metabolomic Analyses of HB1628

*Bifidobacterium bifidum* B1628 was isolated from the fecal sample of a healthy infant and stored in the China General Microbiological Culture Collection Center (CGMCC 22598). The strain was cultured in a reinforced clostridial medium (RCM) at 37 °C for 12 h and then heat-killed at 90 °C for 15 min. The bacterial cells were obtained by centrifugation (4000 rpm/min for 15 min at 4 °C) and washed three times with sterile phosphate buffered saline (PBS). After the washing, the cells were mixed with a protective agent and placed at −80 °C for a pre-freeze. The HB1628 powder was obtained by vacuum freeze-drying and grinding under aseptic conditions. To confirm that the bacteria were inactivated, a small amount of the prepared HB1628 powder was incubated in RCM at 37 °C for 24 h to ensure bacterial non-viability.

After the bacterial suspension was washed with sterile PBS, ultrasound was used to break the cells, and then a UPLC-MS/MS system was utilized to determine the organic acid and short-chain fatty acid contents in the cells. The sample pre-treatment and detection conditions were set according to the previous description in [20]. The statistical analysis and identification of the organic acids and short-chain fatty acids were developed by the R software (accessed on 5 May 2022, http://www.R-project.org/).

### 2.2. Animals and Colitis Model Construction

Specific pathogen-free male C57BL/6J mice (seven-week-old) were purchased from Beijing Huafukang Biotechnology Co., Ltd. (Beijing, China). All of the mice were housed in individually ventilated cages in the animal facility of the Institute of Animal Science of Inner Mongolia Agricultural University under standard conditions (22 °C ± 2 °C, 50% ± 10% humidity, 12 h light/dark cycle). The study was approved by the Animal Welfare and Ethics Committee of Inner Mongolia Agricultural University (NND2022099), and all of the animal handling and experimental procedures were performed in accordance with the National Research Council’s Guide for the Care and Use of Laboratory Animals. 

The mice were acclimatized in the animal facility for 10 days prior to the animal trial. After the acclimatization, they were randomly divided into the following three groups (n = eight per group): the NC group (normal control), the DSS group (induced colitis without postbiotic intervention), and the HB1628 group (induced colitis with postbiotic intervention). Colitis was induced using a 2.5% DSS (MW: from 36,000 to 50,000 Da; MP Biomedicals, LLC, Santa Ana, California, USA). From days 1 to 7, the mice in the DSS and HB1628 groups were given the 2.5% DSS solution instead of regular drinking water. From days 8 to 17, the HB1628 group was given HB1628 (2 × 10^9^ cfu/0.2 mL normal saline/mouse) by oral gavage, while the NC and DSS groups were given the same amount of normal saline. All of the mice were sacrificed on day 18.

### 2.3. Evaluation of Our DSS Model and Histopathological Analysis

Disease activity index (DAI) is the sum of body weight change compared to initial weight, stool consistency, and occult blood. Changes in the DAI were monitored between days 1–7 and on day 18, and the DAI was calculated according to a previous publication with some modifications [21]. 

On day 18, all of the mice were sacrificed. The whole colon lengths were measured, and the intestinal contents were removed and rinsed with phosphate buffered saline before recording the colon weights. The ratio of colon length to weight was calculated to reflect colonic tissue edema. The distal colon (2 cm from the anus) was taken and fixed in 4% paraformaldehyde for histopathological analyses. The tissue samples were embedded in paraffin, sectioned, and stained with hematoxylin and eosin. The stained tissue sections were observed with a light microscope, and the colonic histopathology was scored according to the epithelial cell shedding, connective tissue proliferation, inflammation, and edema. The criteria were based on our actual results and also on previous publications [22,23].

The detailed scoring rules for assessing the DAI and colonic histopathology are shown in Table S1.

### 2.4. Luminex Multiplex Cytokine Assay

The serum cytokines (IL-1β, IL-6, IL-10, IL-13, and TNF-α) were analyzed using the ProcartaPlex^TM^ immunoassay kit (eBioscience, Paris, France), according to the manufacturer’s instructions. The cytokine levels were read on a MAGPIX^®^-Luminex instrument (Merck Millipore, Darmstadt, Germany). The ProcartaPlex Analyst 1.0 software was used to calculate the corresponding cytokine levels from the standard curves. The data were expressed in pg/mL.

### 2.5. DNA Extraction, Metagenomic Sequencing, and Bioinformatics Analysis

For the collection of the mouse fecal samples, each mouse was placed separately in a vacuum steam-sterilized mouse cage with no bedding. The mouse was allowed to defecate naturally, and the feces were collected in sterile 1.5 mL Eppendorf tubes and stored at −80 °C. One gram of the thawed fecal samples was used for DNA extraction using the QIAamp Stool Mini Kit (QIAGEN, Hilden, Germany). The NEBNext^®^ Ultra™ DNA Library Prep Kit (New England Biolabs, Inc., Ipswich, MA, USA) was used to construct sequencing libraries, according to the manufacturer’s instructions. Complete paired-end metagenome sequencing was performed on an Illumina NovaSeq 6000 platform. The sequence data were quality controlled and processed by the KneadData pipeline (accessed on 2 June 2022, http://huttenhower.sph.harvard.edu/kneaddata; v0.7.5). Then, species annotation and functional metagenomic analyses were carried out through the Humann2 pipeline [24].

### 2.6. Statistical Analysis

The results were expressed as mean ± standard deviation. The statistical differences in the mice’s body weight, DAIs, histology scores, colon lengths/weights, and cytokines between the groups were determined by a one-way ANOVA and a Tukey post-hoc tests. The graphical visualization was achieved by Origin 2017. Wilcoxon tests and the R software (version 4.1.1) were used to analyze the statistical differences between the groups and generate a graphic presentation of the metagenomic data, respectively. Alpha and beta diversity (Bray–Curtis distance) analyses were performed using the R package vegan. The correlation between the clinical parameters and the differential gut microbiota was calculated using the Spearman’s rank correlation coefficient, and *p* < 0.05 was considered statistically significant.

## 3. Results

### 3.1. Organic Acids and Short-Chain Fatty Acids in HB1628 Detected by Targeted Metabonomics

**Figure 1:**
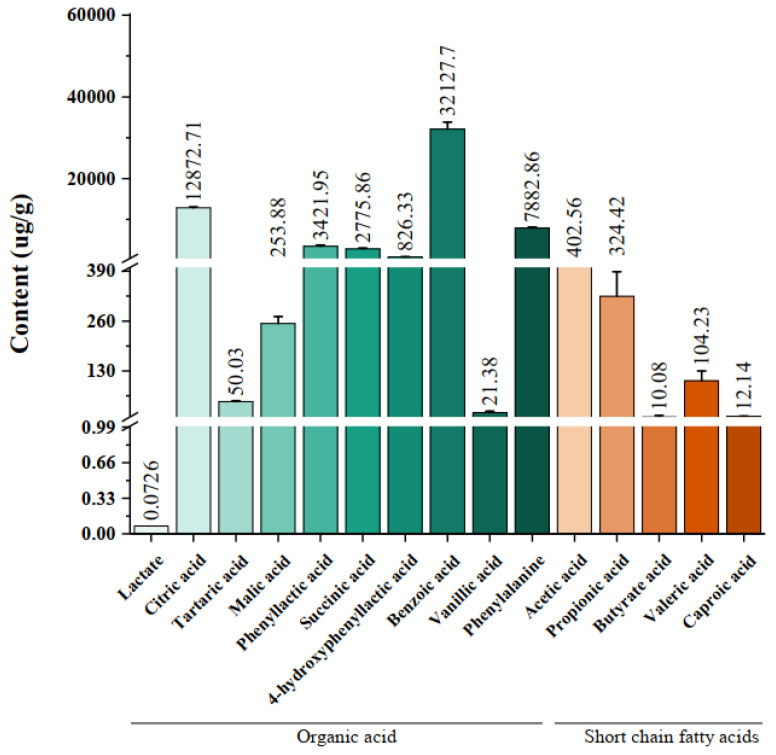
Organic acids and short-chain fatty acids in heat-killed *Bifidobacterium bifidum* B1628. The green gradient represents different kinds of organic acids, and the red gradient represents different kinds of short-chain fatty acids. The error bars represent standard deviations for the five determinations.

The categories and contents of the organic acids and short-chain fatty acids in HB1628 are shown in Figure 1. Ten organic acids were detected, including lactate (0.0726 ± 0.00), citric acid (12872.71 ± 211.9), tartaric acid (50.03 ± 2.49), malic acid (253.88 ± 17.60), phenyllactic acid (3421.95 ± 296.78), succinic acid (2775.86 ± 264.70), 4-hydroxyphenyllactic acid (826.33 ± 41.88), benzoic acid (32127.70 ± 1675.55), vanillic acid (21.38 ± 4.11), and phenylalanine (7882.86 ± 242.52). The contents of benzoic acid and citric acid were the highest. Five short-chain fatty acids were found, which were acetic acid (402.56 ± 40.09), propionic acid (324.42 ± 64.46), butyric acid (10.08 ± 3.80), valeric acid (104.23 ± 26.26), and caproic acid (12.14 ± 0.63), among which acetic acid and propionic acid were the dominant ones.

### 3.2. HB1628 Administration Attenuated DSS-Induced Colitis in Mice

**Figure 2:**
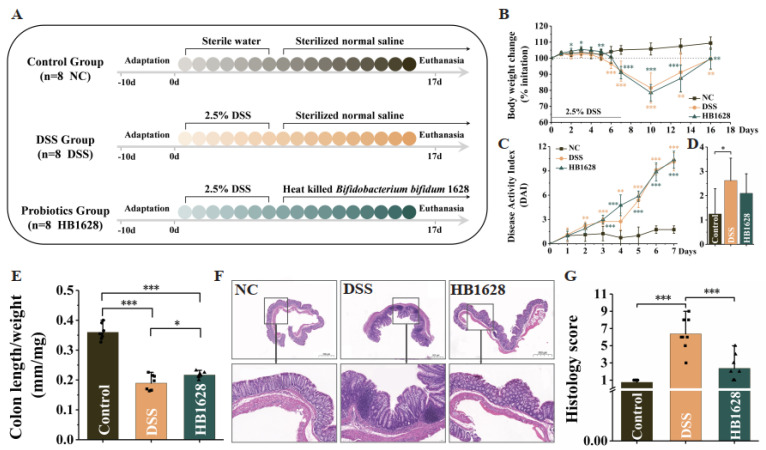
Heat-killed *Bifidobacterium bifidum* B1628 attenuated colonic damage in dextran sulfate sodium (DSS)-induced colitis. (**A**) Experimental design. (**B**) Daily body weight change. (**C**,**D**) Disease activity index during the DSS induction and at day 18. (**E**) Colon length/weight ratio. (**F**) Representative hematoxylin- and eosin-stained micrographs of colon tissue sections (at 20× and 200× magnification) at day 18. (**G**) Histology score. Control group (NC): without DSS induction; DSS group (DSS): 2.5% DSS induction; HB1628 group (HB1628): 2.5% DSS and 0.2 mL of *Bifidobacterium bifidum* B1628 (n = eight per group; * represents *p* < 0.05, ** represents *p* < 0.01, *** represents *p* < 0.001). The error bars represent the standard deviation.

The mice were treated with HB1628 for ten days to ascertain its ameliorative effect on colitis (Figure 2A). The intake of DSS significantly impeded the weight gain of the mice in the DSS and HB1628 groups. The weight loss effect continued after stopping the DSS intake until day 10, when this trend started to reverse (Figure 2B). The DAI, which is an indicator of colitis, rose during the entire period of DSS induction (Figure 2C). At day 18, the value of DAI in the DSS group was significantly higher than that of the NC group (*p* < 0.05; Figure 2D), and the HB1628 intervention reduced the DAI, though non-significantly (*p* > 0.05; Figure 2D). At day 18, the colon length/weight ratio of both the DSS and HB1628 groups was significantly smaller than that of the NC group (*p* < 0.001; Figure 2E), but it was significantly larger in the HB1628 group compared with the DSS group (*p* < 0.05; Figure 2E). The gut tissue of the NC group was healthy, in contrast to the severe induced inflammatory infiltration, epithelial cell damage, and tissue edema in the DSS group. The severity of the inflammation, tissue damage, and edema in the HB1628 group was obviously lower than that in the DSS group (Figure 2F). Thus, the histology score of the DSS group was significantly higher than that of the NC and HB1628 groups (*p* < 0.001; Figure 2G), but no significant differences were observed between the NC and HB1628 groups (*p* > 0.05; Figure 2G). These results together suggested that DSS induced colitis in the DSS-treated mice and that the HB1628 administration mitigated the severity of the colitis. 

### 3.3. HB1628 Administration Regulated Serum Cytokine Levels

**Figure 3:**
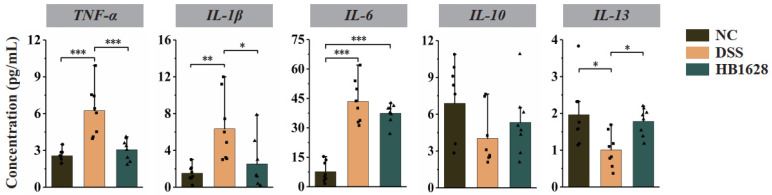
Serum cytokine levels of the three mouse groups at day 18. The serum concentrations of five cytokines were determined, i.e., tumor necrosis factor-α (TNF-α), interleukin (IL)-1β, IL-6, IL-10, and IL-13. Control group (NC): without DSS induction; DSS group (DSS): 2.5% DSS induction; HB1628 group (HB1628): 2.5% DSS and 0.2 mL of *Bifidobacterium bifidum* B1628 (n = eight per group; * represents *p* < 0.05, ** represents *p* < 0.01, *** represents *p* < 0.001).

Cytokines are soluble proteins produced by immune and immune-related cells to maintain immune homeostasis and respond to external insults, such as infections, which reflect the inflammatory state of the mice. Thus, the serum cytokine levels in the three mouse groups were determined at day 18 (Figure 3). The levels of pro-inflammatory cytokines (TNF-α, IL-1β, and IL-6) in the DSS group were significantly higher than those of the NC group (*p* < 0.01; Figure 3), and the anti-inflammatory cytokine IL-13 showed an opposite trend. Compared with the DSS group, the HB1628 group showed significantly lower levels of TNF-α and IL-1β (*p* < 0.05) and a non-significantly reduced levels of IL-6 (*p* > 0.05), but a significantly higher levels of IL-13 (*p* < 0.05). Although not significant, the trend of changes in the anti-inflammatory cytokine IL-10 was similar to that of IL-13. These results together suggested that the DSS treatment caused colon inflammatory responses, and the colon inflammation was largely mitigated by the HB1628 intervention.

### 3.4. HB1628 Administration Modulated the Gut Microbiota Structure

**Figure 4:**
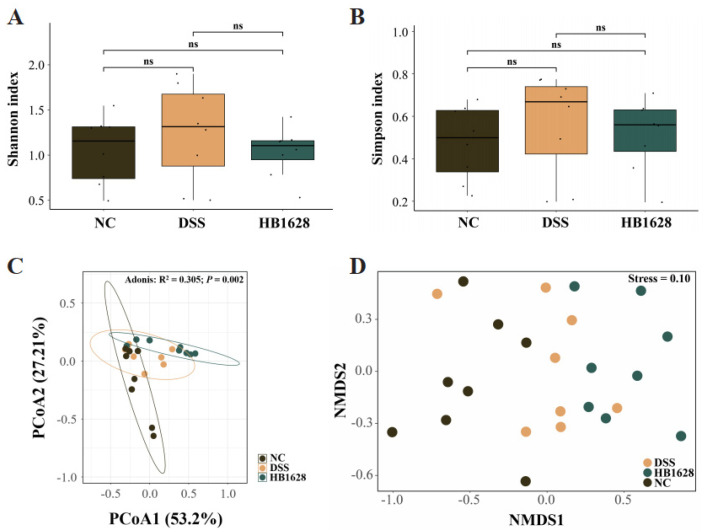
Diversity of gut microbiota in the three mouse groups at day 18. (**A**) Shannon index. (**B**) Simpson index. (**C**) Principal coordinate analysis (PCoA, Bray–Curtis distance) and (**D**) non-metric multidimensional scaling (NMDS, Bray–Curtis distance). Control group (NC): without DSS induction; DSS group (DSS): 2.5% DSS induction; HB1628 group (HB1628): 2.5% DSS and 0.2 mL of *Bifidobacterium bifidum* B1628 (n = eight per group; “ns” means not significant).

Next-generation metagenomic sequencing was applied to characterize the diversity and composition of the mouse gut microbiota. No significant differences were found in the Shannon and Simpson indices among the NC, DSS, and HB1628 groups (*p* < 0.05; Figure 4A,B). Then, the beta diversity of the mouse gut microbiota was analyzed by a principal coordinate analysis (Bray–Curtis distance), which showed that the fecal microbial landscape of the NC, DSS, and HB1628 groups was significantly different (Adonis test: R^2^ = 0.305, *p* = 0.002; Figure 4C). This result was further confirmed by a non-metric multidimensional scaling analysis. Although the symbols representing the three sample groups showed mild overlapping in the score plot, the group-based distribution trends were obvious (stress = 0.10; Figure 4D).

### 3.5. Family-, Genus-, and Species-Level Differences in the Gut Microbiota between Groups

**Figure 5:**
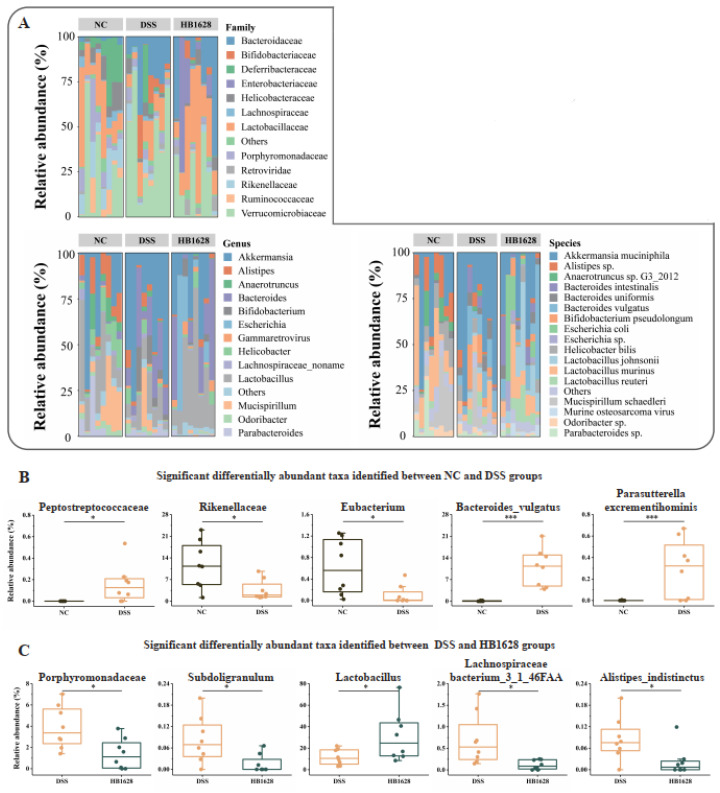
Taxonomic distribution of mouse gut microbiota and significant differentially abundant gut microbes. (**A**) Family-, genus-, and species-level distributions of the mouse gut microbiota. Significant differentially abundant gut bacterial families, genera, and species between the (**B**) NC and DSS groups and the (**C**) DSS and HB1628 groups. Control group (NC): without DSS induction; DSS group (DSS): 2.5% DSS induction; HB1628 group (HB1628): 2.5% DSS and 0.2 mL of *Bifidobacterium bifidum* B1628 (n = eight per group; * represents *p* < 0.05, *** represents *p* < 0.001).

We then identified the top taxa and differentially abundant families, genera, and species with an overall average relative abundance of >1% of the complete gut microbiota dataset (Figure 5A,C; Tables S2–S4). At the family level, *Verrucomicrobiaceae* and *Lactobacillaceae* were the dominant families in the gut microbiota across all of the three mouse groups, implicating that these microorganisms are the intestinal resident bacteria important for maintaining a stable intestinal barrier. More *Bacteroidaceae* were found in the DSS and HB1628 groups than in the NC group (18.47% in the DSS group, 20.90% in the HB1628 group, and 1.44% in the NC group). The family- and genus-level taxonomic distributions were consistent. *Akkermansia* (which belongs to *Verrucomicrobiaceae*) and *Lactobacillus* (which belongs to *Lactobacillaceae*) were the dominant genera across all of the groups. However, more *Akkermansia* were detected in the DSS group than in the other two groups (42.60% in the DSS group, 21.98% in the NC group, and 19.34% in the HB1628 group), while the HB1628 group had the most *Lactobacillus* (30.93% in the HB1628 group, 21.99% in the NC group, and 11.73% in the DSS group). At the species level, *Akkermansia muciniphila* and *Ligilactobacillus murinus* were the top two dominant species in the NC and DSS groups, respectively, but in the HB1628 group, *Akkermansia muciniphila* and *Lactobacillus johnsonii* were the most abundant ones (Figure 5A, Tables S2–S4).

The significant differences between the groups (NC group versus DSS group; DSS group versus HB1628 group) were further analyzed to explore the specific effects of DSS and HB1628 on the individual gut microbes. Significantly fewer *Rikenellaceae* and *Eubacterium* and significantly more *Peptostreptococcaceae*, *Bacteroides vulgatus*, and *Parasutterella excrementihominis* were present in the DSS group compared with the NC group (*p* < 0.05; Figure 5B); these bacteria might be related to the elevated intestinal inflammation after the DSS treatment. 

Significantly more *Lactobacillus* was found in the HB1628 group compared with the DSS group (*p* < 0.05). Meanwhile, significantly fewer *Porphyromonadaceae*, *Subdoligranulum*, *Lachnospiraceae bacterium* 3_1_46FAA, and *Alistipes indistinctus* were detected in the HB1628 group compared with the DSS group (*p* < 0.05; Figure 5C).

### 3.6. Differences in Gut Microbial Metagenomic Potential between the DSS and HB1628 Groups

**Figure 6:**
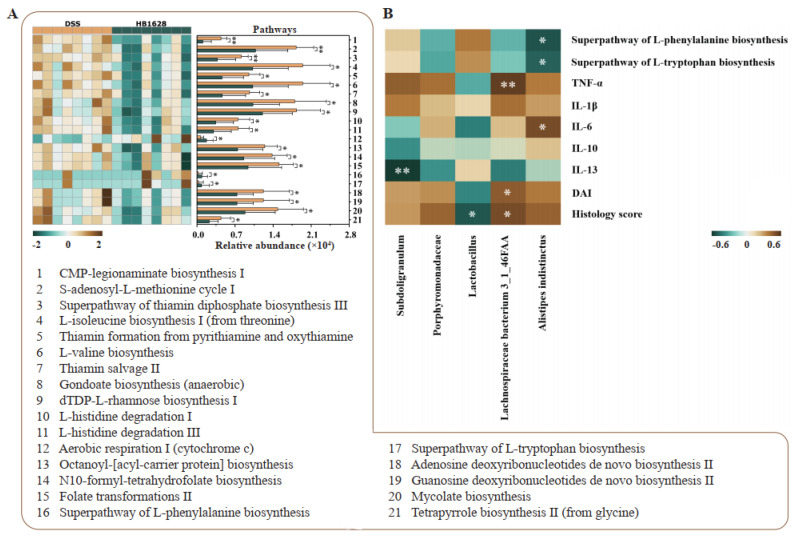
Gut metagenomic potential and correlation analyses. (**A**) Significant differences in the gut metagenomic potentials between the DSS (colitis) and HB1628 (colitis and postbiotic intervention) groups (n = eight per group). The color scale represents the relative abundance of specific pathways encoded in the fecal metagenome (ranging from a high relative abundance [2; dark brown] to a low relative abundance [−2; dark green]). The error bars represent the standard deviation. (**B**) Correlation heat map of the Spearman’s rank correlation coefficient between the differentially abundant taxa and various parameters. The color scale represents the strength of the correlation coefficient (ranging from 0.6 [brown, a strong positive correlation] to −0.6 [green, a strong negative correlation]). * represents *p* < 0.05; ** represents *p* < 0.01. TNF = tumor necrosis factor; IL = interleukin; DAI = disease activity index.

Significant differential pathways encoded in the gut microbial metagenomes between the DSS and HB1628 groups were identified to evaluate the differences in the functional microbial metagenomic potentials between the two mouse groups (Figure 6A). Overall, 21 differential pathways were identified. Eighteen pathways were significantly enriched in the DSS group, such as the CMP-legionaminate biosynthesis I, S-adenosyl-L-methionine cycle I, thiamin salvage II, gondoate biosynthesis (anaerobic), adenosine deoxyribonucleotides de novo biosynthesis II, guanosine deoxyribonucleotides de novo biosynthesis II, mycolate biosynthesis, etc. In contrast, the remaining three pathways, which are the aerobic respiration I (cytochrome c) pathway, the superpathway of L-phenylalanine biosynthesis, and the superpathway of L-tryptophan biosynthesis, were enriched in the HB1628 group. The different distributions of the differential pathways between the DSS and HB1628 groups suggested potential differences in their metabolic function.

### 3.7. Correlation between Differential Gut Microbiota and Clinical Parameters

A correlation heat map was constructed to illustrate the association between the gut microbiota and the clinical parameters (Figure 6B). Some interesting correlations were found. *Lactobacillus* (highly abundant in the HB1628 group) showed a significant negative correlation with the histology score (*p* < 0.05, *R* = −0.58). *Lachnospiraceae bacterium* 3_1_46FAA (significantly enriched in the DSS group) showed significant positive correlations with the histology score, the DAI, and the serum TNF-α level (*p* < 0.05, *R* > 0.50). Furthermore, *Subdoligranulum* was significantly and negatively correlated with IL-13 (*p* < 0.01, *R* = −0.71). *Alistipes indistintus* correlated positively with IL-6 (*p* < 0.05, *R* = 0.56) and negatively with the superpathways of L-phenylalanine biosynthesis and L-tryptophan biosynthesis (*p* < 0.05, *R* > 0.50), respectively.

## 4. Discussion

The etiology of IBD is not completely clear, but it is thought that the complex interactions between the host immunity and the gut microbiota play a critical role in the progression of intestinal inflammations. In this study, we aimed to determine the alleviating effects of the postbiotic HB1628 on intestinal inflammation and gut dysbiosis in colitis mice induced by DSS. Our results showed that HB1628 administration could effectively modulate the balance between serum pro-/anti-inflammatory cytokines, reduce colonic tissue damage, and regulate the gut microbiota and its functional potentials; these actions together mitigated the colitis severity.

The safety of administering live bacteria to patients with IBD is still controversial. Alternatively, the use of heat-killed probiotics could minimize such risks or potential side effects of applying live bacteria. Our results suggest that postbiotics prepared from the strain *Bifidobacterium bifidum* B1628 could alleviate colon tissue damage, which is beneficial to remission in colitis mice. The positive effects were likely exerted by the organic acids and short-chain fatty acids in HB1628 by alleviating tissue damage and inflammatory reactions. For example, organic acids could activate the immune system and induce the host’s production of antibacterial peptides and other substances, thus, inhibiting the growth of intestinal pathogens [25]. Short-chain fatty acids have mucosal immunity regulation effects that promote B cell development, regulate T cell differentiation and expansion, and activate inflammasomes [26]. In fact, some previous studies applying heat-killed probiotics showed similar beneficial effects in pre-clinical and clinical trials. For example, heat-killed *Lactiplantibacillus plantarun* Zhang LL could effectively improve the health of colitis rats and alleviate colitis symptoms, including the general morphology and behavior of rats, body weight, stool consistency, and hematochezia [27]. Heat-killed *Lacticaseibacillus casei* Lbs2 lessened the histopathological features of colitis mice by increasing the frequency of FoxP3+ regulatory T cells in mesenteric lymph nodes [28]. Thus, we envisage that postbiotics could be a promising therapeutic strategy for improving colitis. However, there are still limited studies elucidating the mechanisms of postbiotics in mitigating colitis. 

Nowadays, more than a hundred IBD genetic risk loci have been found, including many immune regulatory genes, such as gene-encoding cytokines [29]. The anti-inflammatory cytokines IL-10 and IL-13 are components of tissue homeostasis [30], while the pro-inflammatory cytokines TNF-α and IL-1β have been proven to be the most important pathological mediators of mucosal injuries in IBD [31]. TNF-α and IL-1β are widely involved in local and systemic inflammations [32]. Some microecological agents have been shown to exert remarkable anti-inflammatory activity by regulating pro-/anti-inflammatory mediators/pathways, which are important mechanisms for reducing intestinal inflammation. For example, *Bifidobacterium adolescentis* has shown obvious effects in reducing the levels of the pro-inflammatory cytokines TNF-α, IL-6, IL-1β, IL-18, IL-22, and IL-9, while increasing the anti-inflammatory cytokines IL-10, IL-4, and IL-5 in colon tissue homogenates [33]. Heat-killed *Levilactobacillus brevis* SBC8803 has been shown to inhibit the expression levels of TNF-α, IL-1β, and IL-12, consequently enhancing intestinal epithelial barrier functions and alleviating intestinal inflammation of colonic epithelia when under oxidant stress [34]. Moreover, the same study found that the expression levels of TNF-α, IL-1β, and IL-12 in DSS-treated colitis mice increased drastically and were significantly, though only partly, reversed by transanal administration of heat-killed *Levilactobacillus brevis* SBC8803. Consistently, our study showed that 10 days of HB1628 intervention significantly reduced pro-inflammatory cytokine levels (TNF-α and IL-1β), while it increased anti-inflammatory cytokine levels (IL-13) in the serum. Our histopathology analysis also revealed an accumulation of a large number of immune cells at the inflammatory site and showed that HB1628 application obviously reduced inflammation in the gut tissue, favoring colonic health recovery.

Inflammatory bowel disease is an immune disorder with a poor prognosis that involves multiple cells and molecules. Indeed, it has been postulated that the culprit of IBD stress sensitization is not the excessive inflammatory response of IBD but the triggering of harmful stimuli as a result of gut dysbiosis [35]. As reviewed by Ni and colleagues (2017), any alteration of the gut microbiota could increase the inflammatory response in patients and experimental animals suffering from colitis [36]. Thus, various therapies targeting the host’s gut microbiota have emerged. Differing from probiotics, postbiotics maintain the host’s intestinal homeostasis by enhancing the growth of the endogenous gut microbiota and beneficial bacteria rather than introducing new species to the intrinsic intestinal microbial community [19]. Our results showed that DSS induction significantly reduced the relative abundance of some butyrate-producing bacteria in the fecal microbiota, e.g., *Rikenellaceae* and *Eubacterium*, and significantly increased the relative abundance of some inflammation-related bacteria, such as *Peptostreptococcaceae*, *Parasutterella excrementihominis*, and *Bacteroides vulgatus*, which have been reported to aggravate colitis [37,38,39]. Meanwhile, a high relative abundance of *Akkermansia muciniphila* was detected in the fecal microbiota of the DSS group. *Akkermansia muciniphila* is a double-edged sword in the pathogenesis of IBD. On one hand, it is thought to be an anti-inflammatory species and has even demonstrated beneficial effects in pre-clinical and clinical trials in multiple clinical scenarios [40]. On the other hand, the species has been reported to degrade the mucin and mucus layers of the intestinal mucosa, and its abnormal proliferation could be a pathological factor that aggravates intestinal barrier damage and induces intestinal inflammation [41]. Another study reported that *Akkermansia muciniphila* administration exacerbated the development of colitis-associated colorectal cancer in mice [42]. Zhai et al. (2019) demonstrated the strain-specific anti-inflammatory effects of *Akkermansia muciniphila* on DSS-induced colitis in mice [43]. Therefore, changes in the relative abundance of *Akkermansia muciniphila* in relation to its overall effects on health need to be carefully interpreted. 

The administration of HB1628 was accompanied by significant changes in the gut microbiota composition. One interesting observation was the significantly higher number of *Lactobacillus* in the fecal microbiota of the HB1628-treated DSS colitis mice compared with that of the DSS colitis mice. *Lactobacillus* showed a significant inverse correlation with the histology score and a mild-to-weak negative correlation with the DAI, IL-6, and TNF-α. Generally, *Lactobacillus* is considered a beneficial genus in the gut that plays important physiological roles in health maintenance. For example, many species of this genus secrete antibacterial substances that inhibit intestinal opportunistic pathogens, produce metabolites to regulate intestinal homeostasis, and stimulate the immune system to maintain a gut microbiota balance and normal intestinal barrier functions [44,45,46]. 

The relative abundances of *Porphyromonadaceae* and *Alistipes indistinctus* were higher in the fecal microbiota of the DSS group compared with those of the HB1628 group. *Porphyromonadaceae* is considered a main pathogen in IBD and is linked to intestinal barrier dysfunction and inflammation [47]. The role of *Alistipes indistinctus* in IBD is unclear, but a related species within the same genus, *Alistipes finegoldii*, is a known trigger of intestinal inflammation [48]. In addition, during the clinical diagnosis and treatment of IBD, patients typically suffer not only from gastrointestinal symptoms but also from some mental health problems, including depression, anxiety, and stress [49]. A pre-clinical study that transferred the gut microbiota of colitis mice to germ-free mice induced anxiety-like and desperate behaviors, further confirming the active role of the host’s gut microbiota in driving behavioral changes through intestinal inflammation [50]. We found that the fecal microbiota of the HB1628 group had fewer *Subdoligranulum* and *Lachnospiraceae bacterium* 3_1_46FAA compared with that of the DSS group. Although these two taxa are generally recognized as beneficial bacteria due to their anti-inflammatory properties, *Subdoligranulum* has been reported to be in high abundance in patients with autism [51], and *Lachnospiraceae* was found to be negatively correlated with sleep efficiency and total sleep time [52]. Thus, even though significantly fewer *Subdoligranulum* and *Lachnospiraceae bacterium* 3_1_46FAA were found in the fecal microbiota of the mice that underwent the 10-day HB1628 intervention compared with those in the DSS group, this might not necessarily be an adverse effect.

As HB1628 mitigates DSS-induced gut dysbiosis by enhancing taxa that are generally thought to be beneficial to the host (particularly *Lactobacillus*) while inhibiting potentially harmful ones, the functional metagenomic potential might also be modulated. Thus, we then identified differential metabolic pathways between the HB1628 and DSS groups. Our analysis returned two interesting and significantly different abundant pathways, i.e., the superpathways of L-tryptophan biosynthesis and L-phenylalanine biosynthesis. Both pathways are related to amino acid synthesis, and both of them were significantly enriched in the HB1628 group compared with the DSS group. Decreased tryptophan metabolic levels are associated with impaired intestinal epithelial barriers in IBD, and some gut microbes could convert tryptophan into bioactive molecules, activate aromatic hydrocarbon receptors, and lower inflammation [5]. Thus, insufficient tryptophan levels reduce the microbe-originated anti-inflammatory effects. Furthermore, tryptophan is the precursor of 5-hydroxytryptamine, which is an important neurotransmitter in the human body. It shows anti-depressive effects and can improve sleep quality and analgesia [53]. L-phenylalanine is an essential aromatic amino acid that has been confirmed to have an anti-inflammatory role in inflammation development [54]. Similar to 5-hydroxytryptamine, L-phenylalanine has been shown to effectively relieve mental issues, such as depression and anxiety. Thus, the enrichment of the biosynthesis pathways of tryptophan and L-phenylalanine is one of the desirable effects after an HB1628 administration.

In conclusion, this study showed that HB1628 is a postbiotic candidate with a high application potential for alleviating colon tissue damage, inflammation, and other symptoms associated with IBD. The symptom mitigation effect was accompanied by significant changes in the host’s serum cytokine profile, gut microbiota, and its encoded functional potential. Our findings have provided proof of principle for the effectiveness of HB1628 administration in mitigating colitis symptoms in a mouse model. This opens up a prospect for the future use of postbiotics to manipulate the microbiome of patients and merits further investigation of the functional effect of HB1628 in managing IBD in human subjects. However, the specific effective substances in the postbiotic HB1628 are still unclear; thus, more detailed isolation and verification are needed to explore the mechanism of HB1628 in alleviating colitis. In the future, more precision research should be carried out on specific postbiotics prepared by different strains.

## Data Availability

Not applicable.

## Supplementary Materials

The following supporting information can be downloaded at: https://www.mdpi.com/article/10.3390/nu14245233/s1, Table S1. Scoring rules for the disease activity index and histology score. Table S2. Gut microbiota of normal, colitis, and postbiotic-fed colitis mice at the family level. Table S3. Gut microbiota of normal, colitis, and postbiotic-fed colitis mice at the genus level. Table S4. Gut microbiota of normal, colitis, and postbiotic-fed colitis mice at the species level.
